# Increases in housing rules and surveillance during COVID-19: impacts on overdose and overdose response in a community-based cohort of sex workers who use drugs in Vancouver, BC

**DOI:** 10.1186/s12954-024-01030-w

**Published:** 2024-08-22

**Authors:** Jenn McDermid, Jennie Pearson, Melissa Braschel, Sarah Moreheart, Rory Marck, Kate Shannon, Andrea Krüsi, Shira M. Goldenberg

**Affiliations:** 1 Centre for Gender and Sexual Health Equity [CGSHE], University of British Columbia Faculty of Medicine, 1081 Burrard Street, Vancouver, BC V6Z 1Y6 Canada; 2 School of Public Policy, Simon Fraser University, Burnaby, BC Canada; 3 Faculty of Health Sciences, Simon Fraser University, Burnaby, BC Canada; 4 Community Scholars Program, Simon Fraser University, Burnaby, BC Canada; 5 Department of Medicine, University of British Columbia, Vancouver, BC Canada; 6 SDSU/UCSD Joint Doctoral Program in Public Health (Epidemiology), Division of Epidemiology and Biostatistics, School of Public Health, San Diego State University (SDSU), San Diego, CA USA

**Keywords:** Surveillance, Housing, Criminalization, Gender, Sex work, Drug use, COVID-19, Unregulated drug toxicity crisis

## Abstract

**Introduction:**

Since the beginning of the COVID-19 pandemic, COVID-19 risk mitigation measures have expanded to include increased rules and surveillance in supportive housing. Yet, in the context of the dual public health emergencies of COVID-19 and the unregulated drug toxicity crisis, we have not evaluated the unintended health and social consequences of such measures, especially on criminalized women. In order to address this dearth of evidence, our aim was to assess the association between increased housing rules and surveillance during COVID-19 and (a) nonfatal overdose, and (b) administration of naloxone for overdose reversal among women sex workers who use drugs in Vancouver, BC.

**Methods:**

This study is nested within An Evaluation of Sex Workers Health Access (AESHA), a community-based prospective cohort of women sex workers in Metro Vancouver (2010–present). Using cross-sectional data collected during the first year of COVID-19 (April 2020–2021), we developed separate multivariable logistic regression confounder models to examine the independent associations between experiencing increased housing rules and surveillance during COVID-19 on (a) nonfatal overdose, and (b) administration of naloxone for overdose reversal in the last 6 months.

**Results:**

Amongst 166 participants, 10.8% reported experiencing a recent non-fatal overdose and 31.3% recently administered naloxone for overdose reversal. 56.6% reported experiencing increased rules and surveillance within their housing during COVID-19. The prevalence of non-fatal overdose and administering naloxone was significantly elevated among those exposed to increased housing rules and surveillance during COVID-19 versus those who were unexposed (83.3% vs. 52.1%; 75.0% vs. 48.2%, respectively). In separate multivariate confounder models, exposure to increased housing rules and surveillance during COVID-19 was independently associated with increased odds of administering naloxone [AOR: 3.66, CI: 1.63–8.21], and marginally associated with non-fatal overdose [AOR: 3.49, CI: 0.92–13.27].

**Conclusion:**

Efforts to prioritize the right to safe, adequate and affordable housing must avoid reinforcing an overly coercive reliance on surveillance measures which, while often well-intended, can negatively shape residents’ well-being. Furthermore, public health responses to pandemics must include criminalized populations so that measures do not exacerbate overdose risk. Implementation of a regulated drug supply is recommended, alongside housing policies that promote residents' rights, safety, and health.

## Introduction

Over the last 3 years, criminalized women, including sex workers and people who use drugs (PWUD), have been disproportionately impacted by the dual public health emergencies of COVID-19 and the unregulated drug toxicity crisis [1, 2]. Past evidence has demonstrated that, in general, sex workers who use drugs experience heightened targeting, harassment and surveillance, due in part to their overlapping identities as sex workers and drug users, both of which are highly criminalized and stigmatized. Such overlapping criminalization has been shown to add an additional layer of overdose risk, resulting in pronounced health inequities and exposure to workplace violence [1, 3–5]. Additional work has illustrated the ways in which the overdose crisis is gendered, with factors such as gender power differentials within drug scenes, housing precarity, and stigma contributing to gendered differences in drug use experiences, and increasing vulnerability to adverse outcomes including violence and overdose [6–8].While it is important to emphasize that drug use is not synonymous with sex work, COVID-19 has further exacerbated many of the structural harms faced by women who both use drugs and engage in sex work, especially in the context of the ongoing unregulated drug toxicity crisis [2].

There is a substantial body of evidence showing that responses to health and social inequities that involve the use of tactics such as surveillance and coercive control are detrimental to the public health of criminalized communities [9–11]. Despite this evidence, since the beginning of the COVID-19 pandemic, pandemic response measures such as physical isolation and the closure of harm reduction sites and other health services, combined with increased rules and restrictions have disproportionately infringed on the health and rights of criminalized communities [12], with sex workers who use drugs especially affected. Particularly, sex workers and PWUD have been increasingly exposed to systems of surveillance within supportive housing in British Columbia (BC) [13], with minimal evaluation done to determine the unintended health and social consequences of such measures, especially in the context of the ongoing unregulated drug toxicity crisis. Within our study, supportive housing is defined as subsidized housing with on-site supports that provide affordable housing and services to low-income adults and families [14]. While some elements of surveillance have almost always been in place in supportive housing, including the use of physical surveillance technologies, site-specific modes of coercion, collaborative relationships with police, and staff surveillance (e.g., guest logs, unannounced room inspections), [15, 16] substantial changes in building policies and rules as COVID-19 risk mitigation strategies resulted in the installation of additional locks and doors, increased monitoring by building staff, enforced curfews, the closure of shared spaces, and stringent guest restrictions [13]. Although these measures were implemented to reflect public health directives, such increases in housing rules and surveillance may further transform supportive housing into sites of social control, leading to isolation and frustration, limiting access to communal spaces and services, reducing resident autonomy, exacerbating experiences of stigmatization and criminalization, and placing women and gender minorities at increased risk of violence [15–17]. Evidence suggests that people who are criminalized in particular can experience considerable harm as a result of surveillance, indicating that women who use drugs and engage in sex work may be especially vulnerable to the negative impacts associated with the COVID-19 related increases in housing rules and surveillance, including heightened risk of overdose [15, 17–21].

With an increasingly unpredictable unregulated drug supply and escalation in overall substance use across the province, the unregulated drug toxicity crisis accelerated in BC during the COVID-19 pandemic [12]. By the end of 2020, the BC Coroner Service had reported 1716 unregulated drug toxicity deaths—the largest single year-over-year increase at that point since 2010 [22]. Between 2019 and 2020 overdose deaths in supportive housing in particular increased considerably, with deaths in the first half of 2020 (January–August) doubling the number of overdose deaths that occurred in supportive housing in all of 2019 [22, 23]. Many suggested that this trend was due, in part, to pandemic guidelines that excluded and neglected the needs of PWUD [12, 24, 25]. For example, BC’s public health orders regarding physical distancing were contradictory to instructions given to PWUD about never using drugs alone—even directives to never use drugs alone however, which, while useful given the unpredictable nature of the current drug supply, do not acknowledge the social and structural contexts of the unregulated drug toxicity crisis and skirts government responsibility in mitigating the upstream drivers of the crisis (e.g., prohibition). Work by Tyndall and Dodd [26] highlights the problem with such inconsistencies in public health messaging, pointing to the fact that social arrangements, including the criminalization of drug use and sex work, isolation, surveillance, and restricted harm reduction access, foster an exceedingly dangerous environment for PWUD in the context of COVID-19 and the unregulated drug toxicity crisis [26].

Compared with other jurisdictions across North America, Vancouver, BC has been at the forefront of implementing overdose-related interventions to address the ongoing unregulated drug toxicity crisis. As an example, in response to the unregulated drug toxicity crisis, naloxone programs have rapidly expanded within supportive housing with residents having easy and free access to naloxone [27–29]. Research has indicated that, with the availability of naloxone, residents in diverse housing environments have been able to take care of their own overdose prevention needs, mitigating accessibility barriers such as stigma, restrictive housing policies and limited hours and access to harm reduction and health services [27, 28, 30]. The expansion of naloxone programs within housing, including programs that facilitate PWUD-led approaches to naloxone training, distribution and overdose response (e.g., the Tenant Overdose Response Organizers in Vancouver), have been shown to be highly effective in reducing the number of fatal overdoses, and enabling PWUD to quickly attend to an overdose [27, 28, 31–34]. While naloxone is a critical life-saving intervention that should be made easily available across jurisdictions, some have suggested that naloxone distribution within supportive housing as a sole response to the unregulated drug toxicity crisis is insufficient as it places the onus of attending to an overdose onto individuals who may not be ready or able to administer naloxone—particularly those who are using alone. Rather, given the unpredictability of the unregulated drug supply, more robust structural interventions are also needed to respond to such a massive and ongoing loss of life [26, 29].

While past research has drawn important connections between supportive housing and the health, safety and well-being of residents [18, 28, 35, 36], what remains underexplored in the context of COVID-19 and the unregulated drug toxicity crisis is the impact that increased supportive housing rules and surveillance have on criminalized women, including sex workers and PWUD. Structural violence [37] provides a useful conceptual lens for examining the ways in which surveillance within supportive housing shape the experiences of women sex workers who use drugs. Structural violence refers to how social structures and institutions sustain, perpetuate, and normalize inequalities and resulting harms [38]. For example, analyses applying this lens have highlighted how macrocontexts (e.g., drug prohibition, criminalization, enforcement) have an impact on microcontexts (e.g., drug use practices), shaping the health and safety of PWUD [26, 38].

Using this analytical framework, we evaluated the overdose and overdose response experiences of criminalized women during the COVID-19 pandemic in relation to changes in housing rules and surveillance within indoor housing environments, where most overdoses occur [39]. While supportive housing remains one of the only forms of urban housing accessible to low-income women who use unregulated drugs, we know little about how increasing housing rules and surveillance during COVID-19 impact overdose risk and the need among residents to engage in overdose response efforts amidst the ongoing unregulated drug toxicity crisis. Our aim was to assess the independent association between increased housing rules and surveillance during COVID-19 and outcomes of (a) nonfatal overdose, and (b) naloxone administration for overdose reversal in the last 6 months amongst sex workers who use drugs in Vancouver, BC.

## Methods

### Study design

Data were drawn from AESHA (An Evaluation of Sex Workers’ Health Access), a community-based longitudinal, cohort study which investigates the impact of social and structural factors on sex workers safety, health and human rights. AESHA has built on collaborations with sex work organizations and has included experiential staff (current/former sex workers) on the project team since inception in January 2010. AESHA’s work continues to be guided by a community advisory board made up representatives across over 15 organizations [40].

Building on the project’s community connections, participant recruitment takes place online (e.g., placing advertisements online) or through outreach to outdoor (e.g., street) and indoor sex work locations (e.g., massage parlours, in-call locations) across Metro Vancouver. Recruitment criteria include having exchanged sex for money within the last 30 days, providing informed consent, and identifying a cis or trans woman (inclusive of diverse self-reported transfeminine identities at enrolment).^1^

The AESHA questionnaire is administered in English, Cantonese or Mandarin at baseline and semiannual follow-up visits either in person or over the phone via the University of British Columbia’s secure REDCap platform by a trained interviewer. This study drew on cross-sectional questionnaire data on socio-demographics, structural factors, health access and outcomes, as well as a COVID-19 supplementary questionnaire implemented in April 2020 that included self-reported impacts of the pandemic on housing and income; work environment; safety; and social outcomes.

All participants received $60 CAD at each biannual visit for their time and expertise. The study holds ethical approval through the Providence Health Care/University of British Columbia Research Ethics Board.

### Study variables

For this study, the team built on their decades-long involvement in community (e.g., frontline harm reduction and social service work, advocacy, community organizing, etc.), current/former sex work experience, and experience working closely with sex workers who use drugs to guide analysis. Drawing also on the considerable loss experienced among the team as a result of the unregulated drug toxicity crisis (e.g., including the loss of family, friends, colleagues, etc.), focus was placed on the myriad systems failures that have allowed over 14,000 people to die from overdose in BC.

Our *primary exposure of interest* assessed whether participants were exposed to increased housing rules or surveillance during the COVID-19 pandemic. This was based on the question “have you experienced any changes related to your housing since the beginning of the COVID-19 pandemic?” that was developed based on community input and piloted in spring 2020. Responses in which a participant selected one or more of the following were coded as ‘yes’: changes to housing policies (e.g., guest restrictions, reduced access to communal spaces) and increased surveillance in their building (e.g., increased monitoring by building staff and security guards, enforced curfews) or relevant ‘other’ (e.g., installation of additional building locks, doors). Participants who responded that they had not experienced any increase in housing rules or surveillance were coded as ‘no’.

Two outcome variables were used in this analysis: (a) nonfatal overdose, defined as responding “yes” to the question, “In the last 6 months, have you overdosed by accident?”; and (b) engagement in naloxone administration, defined as responding “yes” to the question, “Have you administered naloxone to anyone in the last 6 months?”.

Independent demographic and structural variables were considered as potential confounders, and were identified based on a literature review related to drug use, surveillance and housing, and informed by bivariate analyses. *Demographic variables* included Indigeneity (i.e., First Nations, Métis, Inuit), sexual minority identity (i.e., identifying as gay, lesbian, bisexual, asexual, queer, other), age (years), currently living in Vancouver’s Downtown Eastside, and any diagnosis, treatment, or support for depression, anxiety, or PTSD in the last 6 months. *Living environment* variables included living in supportive housing or a shelter in the last 6 months and feeling in danger where currently sleeping. *Drug use & drug safety* variables were selected based on documented associations between types and methods of drug use, and experiences of a non-fatal overdose [4]. In light of concerns related to an unpredictable and unregulated drug supply, and results from our bivariate analyses, injection and non-injection opioid and stimulant use in the last 6 months were considered as potential confounders. Finally, as participants were asked about changes since the COVID-19 pandemic began in BC in March 2020, interview month was included as an adjustment variable in the confounder models.

### Statistical analyses

Analyses were restricted to study visits where participants reported using criminalized non-injection or injection drugs within the last 6 months and to the sub-sample of cohort participants who we were able to connect with during COVID-19 lockdowns and who completed the COVID-19 supplementary questionnaire from April 2020–April 2021.

Primary exposure and potential confounders of interest were stratified by the two outcomes of interest. We used bivariate logistic regression to examine associations between the primary exposure of interest (increases in COVID-19 related housing surveillance and rules), hypothesized confounders and outcomes of (a) non-fatal overdose, and (b) naloxone administration in the last 6 months. We developed two separate multivariable logistic regression confounder models to model the independent association between experiencing an increase in housing rules or surveillance during COVID-19 on outcomes of (a) non-fatal overdose and (b) administering naloxone to others in the last 6 months. Hypothesized confounders that were significantly associated with the outcome at *p* < 0.15 in bivariate analysis were included in the full model. Using the process described by Maldonado and Greenland [41] potential confounders were removed in a stepwise manner, and variables that altered the association of interest by < 5% were removed from the model. All statistical analyses were performed in SAS version 9.4 (SAS, Cary, NC). Odds ratios (OR), adjusted odds ratios (AOR), and 95% confidence intervals (CI) are presented and all *p* values are two-sided.

## Results

Analyses included 166 sex workers in Metro Vancouver interviewed between April 2020–April 2021 who had used unregulated drugs in the last 6 months.

Over this study covering the first year of the COVID-19 pandemic, 10.8% of participants reported experiencing a recent non-fatal overdose, and 31.3% had administered naloxone in the last 6 months.

Participants reported high levels of concern regarding adverse impacts of public health COVID-19 mitigation measures, the most prominent being increases in housing rules or surveillance, which was reported by 56.6% of participants, among whom both naloxone administration (Table 1) as well as overdose (Table 2) were significantly more prevalent than those not experiencing increased housing rules or surveillance. When restricted to just those living in shelters and supportive housing, this number was even higher with 63.0% of participants reporting increases in housing rules or surveillance. An additional 14.1% of participants reported experiencing ‘other’ housing changes since the onset of COVID-19 (e.g., installation of additional building locks, doors). Over half further reported facing difficulty maintaining a support network as a result of COVID-19 (53.6%), 13.9% reported facing challenges in health and harm reduction service access due to closures or changes in hours of services, and 19.3% reported increasing concerns related to community safety since the beginning of the COVID-19 pandemic.

**Table 1:** Individual and structural characteristics of sex workers who use drugs during COVID-19 in Metro Vancouver, Canada (*n* = 166), stratified by recently administered naloxone, AESHA 2020–2021

Characteristic	Total (%) (*n* = 166)	Outcome: administered naloxone, last 6 months		Odds ratio (95% CI)
		Yes (%) (*n* = 52)	No (%) (*n* = 112)	
*Primary exposure*				
Experienced increased housing rules/surveillance during COVID-19	94 (56.6)	39 (75.0)	54 (48.2)	3.34 (1.61–6.92)
*Demographics and living environments*				
Age (years; median, IQR)	45 (36–52)	45 (39–51)	44 (36–52)	1.01 (0.98–1.05)
Sexual minority	76 (45.8)	25 (15.4)	50 (58.9)	1.15 (0.59–2.22)
Indigenous	89 (53.6)	23 (44.2)	65 (58.0)	0.57 (0.30–1.11)
Lives in the DTES^b^	64 (38.6)	23 (44.2)	41 (36.6)	1.34 (0.68–2.61)
Lives in supportive housing housing^a^	109 (65.7)	39 (75.0)	69 (61.6)	1.87 (0.89–3.89)
Lives in a shelter^a^	25 (15.1)	8 (15.4)	17 (15.2)	1.02 (0.41–2.53)
Feels in danger where currently sleeping	57 (34.3)	27 (51.9)	29 (25.9)	3.09 (1.55–6.16)
Diagnosis/treatment/support for depression/anxiety/PTSD^a^	42 (25.3)	18 (34.6)	24 (21.4)	1.97 (0.95–4.10)
*Substance use patterns*				
Non-injection stimulants^a^	121 (72.9)	41 (78.9)	80 (71.4)	1.44 (0.66–3.16)
Non-injection opioids^a^	68 (41.0)	26 (50.0)	42 (37.5)	1.71 (0.88–3.34)
Injection stimulants^a^	58 (34.9)	23 (44.2)	35 (31.3)	1.70 (0.86–3.35)
Injection opioids^a^	73 (44.0)	26 (50.0)	47 (42.0)	1.34 (0.69–2.59)

All data refer to n (%) of participants unless otherwise specified

^a^In the last 6 months

^b^The Downtown East Side (DTES), a neighbourhood within the City of Vancouver characterized by both social and economic inequities as well as significant community organizing and low-threshold services

**Table 2:** Individual and structural characteristics of sex workers who use drugs during COVID-19 in Metro Vancouver, Canada (*n* = 166), stratified by recent non-fatal overdose AESHA 2020–2021

Characteristic	Total (%) (*n* = 166)	Outcome: non-fatal overdose, last 6 months		Odds ratio (95% CI)
		Yes (%) (*n* = 18)	No (%) (*n* = 144)	
*Primary exposure*				
Experienced increased housing rules/surveillance during COVID-19	94 (56.6)	15 (83.3)	75 (52.1)	4.60 (1.28–16.58)
*Demographics and living environment*				
Age (years; median, IQR)	45 (36–52)	48 (38–50)	44 (36–52)	1.01 (0.96–1.o6)
Sexual minority	76 (45.8)	8 (44.4)	66 (45.8)	0.945 (0.35–2.53)
Indigenous	89 (53.6)	11 (61.1)	76 (52.8)	1.32 (0.49–3.61)
Lives in the DTES^b^	64 (38.6)	8 (44.4)	55 (38.2)	1.27 (0.47–3.40)
Lives in supportive housing^a^	109 (65.7)	14 (77.8)	92 (63.9)	1.98 (0.62–6.32)
Lives in a shelter^a^	25 (15.1)	5 (27.8)	20 (13.9)	2.39 (0.77–7.42)
Feels in danger where currently sleeping	57 (34.3)	10 (55.6)	45 (31.3)	2.75 (1.02–7.43)
Diagnosis/treatment/support for depression/anxiety/PTSD^a^	42 (25.3)	6 (33.3)	36 (25.0)	1.42 (0.50–4.05)
*Substance use patterns*				
Non-injection stimulants^a^	121 (72.9)	16 (88.9)	104 (72.2)	3.08 (0.68–13.98)
Non-injection opioids^a^	68 (41.0)	10 (55.6)	58 (40.3)	1.83 (0.68–4.92)
Injection stimulants^a^	58 (34.9)	11 (61.1)	46 (31.9)	3.31 (1.21–9.10)
Injection opioids^a^	73 (44.0)	14 (77.8)	59 (41.0)	4.98 (1.56–15.90)

All data refer to n (%) of participants unless otherwise specified

^a^In the last 6 months

^b^The Downtown East Side (DTES), a neighbourhood within the City of Vancouver characterized by both social and economic inequities as well as significant community organizing and low-threshold services

With regards to participant demographics, the median age was 45 (IQR: 36–52), 53.6% were of Indigenous (i.e., First Nations, Inuit, Métis) ancestry, 2.4% identified as Black/Women of Colour, and 43.9% identified as white (Table 1). 45.8% identified as a sexual minority. 65.7% had lived in supportive housing in the last 6 months, while 15.1% lived in a shelter and 2.4% lived in an SRO. With respect to drug use, 72.9% had used non-injection stimulants in the last 6 months, while 34.9% used injection stimulants, and 41.0% used non-injection opioids, while 44.0% used injection opioids.

In bivariate analysis, women who faced increased housing rules and surveillance during COVID-19 had higher odds of administering naloxone in the last 6 months (OR 3.34, 95% CI 1.61–6.92) (Table 1). Structural variables including living in supportive housing and feeling unsafe in their current living situation were also associated with increased odds of administering naloxone, as were non-injection opioids (OR 1.71, 95% CI 0.88–3.34) and injection stimulants (OR 1.70, 95% CI 0.86–3.35) use.

Similarly, facing increased housing rules and surveillance during COVID-19 was associated with higher odds of non-fatal overdose in the last 6 months (OR 4.60, 95% CI 1.28–16.58) (Table 2) in bivariate analysis. Structural variables including living in a shelter and feeling unsafe in their current living situation were also associated with increased odds of non-fatal overdose, as were injection opioids (OR 4.98, 95% CI 1.56–15.90) and stimulants (OR 3.31, 95% CI 1.21–9.10) use.

In separate multivariable logistic regression confounder models, exposure to increased housing surveillance and rules in the first year of the COVID-19 pandemic was associated with over three times the odds of administering naloxone (AOR 3.66, 95% CI 1.63–8.21) and was marginally associated with experiencing a non-fatal overdose (AOR 3.49, 95% CI 0.92–13.27, *p* = 0.066) after adjustment for confounders (Table 3). In sensitivity analysis, the positive relationship between exposure to increased housing rules and surveillance and non-fatal overdose remained and was statistically significant when we restricted the sample to only those participants living in shelters and supportive housing.^2^

**Table 3:** Multivariable logistic regression confounder models for the independent association between exposure to increasing housing rules/surveillance during COVID-19 and outcomes of recent non-fatal overdose and naloxone administration amongst sex workers who use drugs in Metro Vancouver, Canada (*n* = 166), AESHA 2020–2021

*Exposure*: Experienced increased housing surveillance and rules in the first year of the COVID-19 pandemic		
Outcomes	Adjusted odds ratio	95% confidence interval
Recent naloxone use	3.66	1.63–8.21
Recent non-fatal overdose	3.49	0.92–13.27

Both models adjusted for month of interview during COVID-19, Indigenous identity, living in the DTES, and injection and non-injection opioid use

## Discussion

Amidst BC’s dual public health emergencies of COVID-19 and the unregulated drug toxicity crisis, this study among criminalized women who engage in both sex work and drug use in Vancouver, BC found that exposure to increased housing rules and surveillance during the first year of the COVID-19 pandemic was associated with increased odds of administering naloxone and marginally associated with increased odds of experiencing non-fatal overdose. Drawing on a structural violence framework, our findings highlight that, while well-intended for the purpose of mitigating the spread of COVID-19, increases in housing rules and surveillance may unintentionally increase overdose risk and require more intensified engagement in subsequent overdose response efforts among residents in the context of the unpredictable, unregulated drug supply, placing a substantial burden on the well-being of women who experience intersecting forms of criminalization (e.g., sex work and drug use).

Amongst sex workers who use drugs, we found that increased housing rules and surveillance during COVID-19 was associated with over 3.5-fold higher odds of administering naloxone for overdose reversal in the past 6 months. This may be attributed to a number of factors including increased distribution of naloxone within supportive housing amidst the ongoing unregulated drug toxicity crisis, pandemic-related isolation, lack of access to harm reduction services, housing staff’s varying levels of comfort in administering naloxone during the first year of the pandemic, and the overlapping criminalization experienced by sex workers who use drugs, reducing the likelihood of calling for help during an overdose and resulting in an increased reliance on personal naloxone use [12, 13, 34]. Additionally, there is heightened potential for those in supportive housing, where housing rules and surveillance are most likely to be experienced, to witness and attend to overdoses fueled by the unpredicable, unregulated drug supply [1]. While research on naloxone programs within supportive housing highlights the critical importance of widespread access to naloxone—for example, by reducing the number of overdose deaths, offering easy access to naloxone for residents, and providing PWUD with the ability to respond quickly to an overdose [28, 32]—naloxone ultimately does not address the ‘upstream’ drivers of overdoses, leaving PWUD at continued risk of critical injury or death [24]. In the absence of structural solutions, reliance on naloxone as a sole response to the unregulated drug toxicity crisis makes it so residents, particularly criminalized residents, within supportive housing are often responsible for keeping themselves and their neighbours safe, even in situations where they may not be ready or able to attend to an overdose (e.g., naloxone is not effective for people who use alone as it cannot be self-administered after an overdose) [30]. While housing policies that facilitate access to a variety of overdose prevention and response tools, including naloxone, are warranted, it is important that more ‘upstream’ overdose prevention programs also be scaled-up.

In addition, our work indicates that exposure to more restrictive housing rules and increased surveillance during the COVID-19 pandemic was marginally associated with 3.49-fold higher odds of experiencing a non-fatal overdose among sex workers who use drugs. In the context of an unpredictable, unregulated drug supply that has become more volatile as a result of the pandemic [24], increased housing rules and surveillance may heighten overdose risk by making it more difficult for residents to employ safer drug use strategies, such as using with others, accessing harm reduction and drug testing services, or buying from trusted sources [17, 24, 42, 43]. Further, qualitative research by Boyd et al. [15] demonstrates that compulsory visibility inside one's home, alongside the enforcement of prohibitory or coercive site-specific rules, may undermine the development and uptake of critical initiatives such as building-run, communal overdose prevention sites, making residents more vulnerable to fatal overdose. Such risks are further exacerbated by gender inequities, criminalization, stigma, and a lack of structural interventions such as the availability of a regulated supply of drugs. We recommend that housing providers carefully assess all policies and rules to ensure that they do not infringe on the human rights of residents—including the ability to practice harm reduction and overdose prevention strategies—which is especially critical among sex workers who use drugs, who face disproportionate overdose risks.

In line with previous evidence [15, 17, 21, 23], findings of this study highlight that people who are criminalized, including sex workers and PWUD, are disproportionately impacted by public health interventions that result in augmented surveillance. While increasing rules and surveillance within supportive housing during COVID-19 was intended to protect residents from illness, in the context of the ongoing unregulated drug toxicity crisis, these measures may also cause unintended negative consequences related to residents’ individual rights and safety. Importantly, we wish to convey that housing rules and surveillance practices are not always inherently punitive or harmful—but rather, these exist on a continuum and can also advance resident safety or provide critical care (e.g., wellness checks that are voluntary and initiated at a resident's request) [21]. In order to avoid unintended negative consequences, it is imperative that the needs, concerns, and experiences of marginalized residents are centred and prioritized as part of any decision-making process.

Some prior research has shown that responses to the COVID-19 pandemic involving heightened surveillance or enforcement measures (including the involvement of police or security personnel) have acted in carceral ways within health and social services, which, rather than providing rights-based structural supports, extend the control that many institutions exert over, criminalized, and racialized populations [17, 19, 20]. Consistent with evidence demonstrating that the use of punitive measures to address health and social inequities are detrimental to public health [9–11], this kind of control has been shown to heighten the harms faced by people who are criminalized [17, 21, 28, 35]. Considering this, where increased housing rules and surveillance have contributed to more dangerous environments for sex workers who use drugs (e.g., due to isolation, increased monitoring, reduced decision-making power/autonomy, mechanisms of social/coercive control), negatively shaping health, well-being and risk of overdose, this can be understood as a form of structural violence [15, 23, 26].

Collectively, our findings highlight the way that enhanced surveillance within supportive housing environments can reinforce—rather than mitigate—the structural vulnerability of sex workers who use drugs, especially in the face of the ongoing unregulated drug toxicity crisis. Although the negative consequences of implementing surveillance tactics in supportive housing during COVID-19 were largely unintended, prior research suggests that sole or primary reliance on restrictive strategies that limit the rights of criminalized women perpetuate unsafe environments [17, 18, 26]. To advance the health and well-being of sex workers who use drugs, responses to public health crises must meaningfully address the structural conditions that place sex workers at increased risk of overdose, including the prioritization of the rights and agency of supportive housing residents. This includes revaluating, reducing or removing changes in housing policies that increase surveillance and transform supportive housing into sites of coercive control (e.g., via unannounced room inspections, restrictive guest policies, police/security involvement in daily housing operations, etc.); providing supportive housing residents with the authority to make decisions about their own space; scaling-up harm reduction services within supportive housing, including supervised consumption areas that support injection and non-injection consumption methods (e.g., injection, snorting, smoking, and ingestion) and that meet the needs of women who use drugs (e.g., gender-specific sites); addressing experiences of unequal gendered power relations within buildings; increasing the different types of dignified affordable housing offered, including alternatives to non-profit managed housing; removing criminal sanctions around drug use and sex work; and, most notably, acting on calls made by PWUD and advocates to provide a safe, accessible and regulated supply of drugs [44, 45].

### Strengths and limitations

This study presents some of the first empirical data on the impacts of increased housing rules and surveillance during the COVID-19 pandemic on the health and safety of women who use drugs, and women sex workers who use drugs specifically, which was informed by community accounts of the harms experienced as a result of such policies. Further longitudinal research is recommended to assess potential policy changes pre-and-post COVID-19 among sex workers who use drugs in supportive housing environments, as this study did not collect pre-COVID data on housing surveillance or policies with which comparisons could be made. Given the challenges of connecting with sex workers throughout COVID-19, and as the original AESHA study was not powered to assess impacts of the COVID-19 pandemic, our restricted sample size may limit statistical power and may have resulted in some imprecision in our estimates. While we drew on cross-sectional data from the semi-annual AESHA questionnaire and a COVID-19 supplementary questionnaire administered with the same participants, there was some variation in reference times for variables between the questionnaires (i.e., in the last 6 months vs. since COVID-19 began in BC in March 2020); a small number of questionnaires were completed up to 3 months apart, which may result in some temporal variation. Although self-reported data are known to be subject to social desirability bias, our frontline staff includes experiential and community-based interviewers with experience in trauma-informed interviewing and building rapport with participants, which has been an effective strategy in mitigating this to the extent possible.

## Conclusion

Efforts to prioritize the right to safe, dignified and affordable housing must avoid reinforcing a reliance on surveillance and coercive measures which, while often well-intended, can restrict individual rights and have deleterious effects on health and overdose outcomes. Our research demonstrated that, amongst sex workers who use drugs in Vancouver, BC, 75.0% of those who had administered naloxone (vs. 48.2% who had not administered naloxone) and 83.3% of those who had experienced an overdose (vs. 52.1% who had not experienced an overdose) were exposed to increases in housing rules and surveillance. In the context of COVID-19 and the unregulated drug toxicity crisis, these findings highlight that intersecting social and environmental forces such as surveillance, unequal gendered power relations, isolation and the criminalization of drug use and sex work can exacerbate the harms of the unpredictable, unregulated drug supply and can contribute to dangerous situations for women who use drugs and who live in supportive housing. Furthermore, public health responses to pandemics need to consider and include criminalized populations to ensure that messaging and responses do not further exacerbate health and social inequities, including risks for unintended overdose.

In order to minimize this risk, our results underscore the urgent need to implement structural interventions, some of which include: providing supportive housing residents with the power to make decisions about their own space and collaboratively manage their building; expanding harm reduction access within supportive housing, including services for injection and non-injection consumption methods and addressing gendered power differentials; increasing the different types of dignified, affordable housing offered, including alternatives to non-profit managed housing; decommodifying housing; ending drug and sex work prohibition; and ensuring access to a safe and regulated supply of drugs [43, 44].

## Data Availability

Due to our ethical and legal requirements related to protecting participant privacy and current ethical institutional approvals, de-identified data are available upon request pending ethical approval. Please submit all request to initiate the data access process to the corresponding author.

## Footnotes

1 Eligibility is intended to capture the ways that patriarchal gender norms shape participants’ experiences. In acknowledging that gender identity can be fluid, we recognize that some participants’ gender presentation differs throughout various aspects of their lives. For example, a participant may present as a woman/femme while interacting with sex work clients but identify as non-binary outside of work environments.

2 Data not shown due to imprecision in estimates resulting from small sample size.
